# Moving fluoroscopy-based analysis of THA kinematics during unrestricted activities of daily living

**DOI:** 10.3389/fbioe.2023.1095845

**Published:** 2023-04-24

**Authors:** Fabio D’Isidoro, Clara Brockmann, Bernd Friesenbichler, Thomas Zumbrunn, Michael Leunig, Stephen J. Ferguson

**Affiliations:** ^1^ Institute for Biomechanics, ETH Zurich, Zurich, Switzerland; ^2^ Schulthess Klinik, Zurich, Switzerland

**Keywords:** hip, kinematics, fluoroscopy, arthroplasty, daily activities

## Abstract

**Introduction:** Knowledge of the accurate *in-vivo* kinematics of total hip arthroplasty (THA) during activities of daily living can potentially improve the *in-vitro* or computational wear and impingement prediction of hip implants. Fluoroscopy- based techniques provide more accurate kinematics compared to skin marker-based motion capture, which is affected by the soft tissue artefact. To date, stationary fluoroscopic machines allowed the measurement of only restricted movements, or only a portion of the whole motion cycle.

**Methods:** In this study, a moving fluoroscopic robot was used to measure the hip joint motion of 15 THA subjects during whole cycles of unrestricted activities of daily living, i.e., overground gait, stair descent, chair rise and putting on socks.

**Results:** The retrieved hip joint motions differed from the standard patterns applied for wear testing, demonstrating that current pre-clinical wear testing procedures do not reflect the experienced *in-vivo* daily motions of THA.

**Discussion:** The measured patient-specific kinematics may be used as input to *in vitro* and computational simulations, in order to investigate how individual motion patterns affect the predicted wear or impingement.

## 1 Introduction

Total hip arthroplasty (THA) is a well-accepted and successful solution for patients with end-stage osteoarthritis, and its demand is expected to rise as demographics reflect an ageing population (Szymanski and Barciszewski, 2017). Despite the positive outcomes (Fortina et al., 2005), the longevity of THA must be improved to counter the expected increase in number of revisions and to meet the needs of a broader and generally more active patient population. With improvements in THA technology and societal expectations for unencumbered life, indications are increasing for arthroplasty in both younger and older subjects. The downturn of metal-on-metal hip prostheses (Hart et al., 2012; Mellon et al., 2013), despite promising technical measures of performance, showed that current *in-vitro* wear testing procedures fail at predicting the true *in-vivo* performance of the hip prosthesis (Scholes and Unsworth, 2006). Comparisons between *ex-vivo* and *in vitro* implant surface textures revealed wider regions of marked wear patterns for retrievals (Manaka et al., 2004; Affatato et al., 2012). These discrepancies were related to differences between *in vitro* and *in vivo* kinematics and kinetics.

Implant wear is currently tested with laboratory simulators, where standardized motion and loading patterns are applied on the hip prosthetic components (ISO 14242-1:2014, 2014). The standard loading pattern consists of a walking profile with a simplified axial loading twin-peak curve, with 3,000 N peak load at “heel strike” and “toe-off,” and a 300 N swing phase. The ISO motion is standardized to +25°/−18° Flexion/Extension, +7°/−4° Adduction/Abduction and +2°/−10° Internal/External rotation, to represent a typical gait pattern 8. Researchers have found that altering the input parameters of wear simulators from the ISO standard can generate clinically relevant changes in wear predictions (Roter et al., 2002; Bowsher et al., 2006; Fabry et al., 2013), and that individual patient activity patterns affect wear of THA (Gao et al., 2011; Mellon et al., 2013).

In addition to laboratory wear testing, there is an increasing shift towards computational simulations of THA tribology and wear, in the interest of improving efficiency, decreasing costs, or exploring broader parameter spaces. Gait driven finite element analyses have been shown to accurately capture experimentally measured wear rates (Toh et al., 2021), and the specific importance of developing appropriate wear coefficients and analysing various daily living activities has been stressed (Wang et al., 2019). Computational wear predictions allow the exploration of unconventional articulating counter-surfaces, in the absence of prohibitive physical prototyping costs (Jamari et al., 2022) and facilitate a full parametric analysis of the contribution of implant geometry, properties and loading conditions on THA contact stresses and wear (Li et al., 2020). The maintenance of low-friction articulation is a requirement for limiting wear, and recently fully coupled simulation models have emerged which allow the prediction of both the evolution of a lubricating fluid film, taking into account implant deformation, and the consequent wear (Gao et al., 2017), and exploring the effect of fluid film depletion in, e.g., ceramic-on-ceramic systems (Meng et al., 2015). Kinematic data has also been used to drive musculoskeletal models, which then provide the boundary conditions for finite element simulations of contact pressure (Affatato et al., 2018).

Impingement, and a corresponding increased risk of joint subluxation, is a further potential cause of implant failure heavily influenced by joint kinematics. Geometry based models, or 3D parametric finite element models, of the implant and bone have been used to evaluate the impingement-free range of motion (ROM) of THA (Alastruey-Lopez et al., 2020; Pryce et al., 2022). Such simulations have provided ROM-to-impingement curves for challenging high flexion and pivot activities (McCarthy et al., 2021). Such predictions allow the pre-operative surgical plan to be adjusted to minimise impingement risk (Palit et al., 2019).

Therefore, the importance of testing the normal function and wear of THA based on motions and loadings from a broader spectrum of activities of daily living (ADL), as well as the need to identify adverse loading scenarios is increasingly acknowledged.

Kinematics and kinetics of THA during different ADLs have been analyzed by Bergmann et al. (2001) in four patients using motion analysis and instrumented prostheses. Skin marker-based motion capture (MC) has been adopted in several studies to measure implanted hip joint motion during gait (Perron et al., 2000; Bennett et al., 2008), stair climbing and descending (Shrader et al., 2009; Lamontagne et al., 2011a), and sitting and standing from a chair (Lamontagne et al., 2012). However, MC-based kinematics are affected by errors due to soft tissue artefact, which is caused by the extensive movement of skin markers relative to the underlying bones (Leardini et al., 2005). The accuracy of MC may not allow to reliably capture kinematic aspects that have been observed to affect wear, such as hip micro separation (Nevelos et al., 2000), prosthetic hip impingement (Malik et al., 2007) and edge loading (Mellon et al., 2013).

Soft tissue artefact-free THA kinematics have been obtained with video-fluoroscopy (VF), a minimally invasive technique where the true motion of the prosthetic components is retrieved directly by 2D/3D registration of implant models with a sequence of X-ray images acquired dynamically during *in-vivo* activities. VF studies have analyzed gait on treadmill (Glaser et al., 2008; Tsai et al., 2013; Tsai et al., 2014), stair climbing (Dimitriou et al., 2015), squatting (Koyanagi et al., 2011), and isolated abduction (Lombardi et al., 2000). However, a constraint of these studies was the limited field of view using a stationary X-ray imaging system (List et al., 2013) that allowed the assessment of only restricted movements, or only a portion of the whole motion cycle. For this reason, gait using VF has only been evaluated on a treadmill. Although similarities in both kinematics and kinetics have been reported between treadmill and overground gait for healthy subjects after an accommodation period (Watt et al., 2010), there is evidence that elderly people may have difficulty accommodating to treadmill walking (Wass et al., 2005). Hence, overground gait is better suited to represent the daily walking of most THA patients.

Moving fluoroscopic systems have been developed in order to measure accurate unrestricted joint motions. ADLs of the prosthetic knee (List et al., 2017) and ankle (List et al., 2012) were analyzed *in-vivo* with moving single-plane fluoroscopy during ADLs. Recently, a moving biplane X-ray imaging system was tested *in-vitro* for total knee arthroplasty during simulated overground gait (Guan et al., 2016).

The present study is the first observational analysis of the *in-vivo* kinematics of THA measured with moving fluoroscopy in a sample population performing whole cycles of both routine and challenging activities of daily living, i.e., overground gait, stair descent, chair rise, and putting on socks.

## 2 Methods

This was an analytic, cross-sectional, observational, non-clinical research study for the collection of health-related data (Level of evidence III).

### 2.1 Subjects and activities

15 subjects with unilateral primary THA due to osteoarthritis (9 men, average age 65 ± 7.4, weight 77.4 kg ± 10.6, height 174.7 cm ± 9.1, BMI 25.3 kg/m^2^ ± 2.2, average follow-up time from surgical date 31 months, cup sizes from 48 to 60 mm, femoral head sizes from 32 to 36 mm, all right implanted side) provided their written informed consent to the study, that was approved by the institutional review board and by the local cantonal ethics committee (BASEC-No. 2016-00438). All patients received a Smith & Nephew Polar Stem and R3 cup with a direct anterior approach, had an Oxford Hip Score >40, none or very low pain (VAS < 2) and standardized general health survey score (SF-12) within the normal range for people in their age group.

**FIGURE 1:**
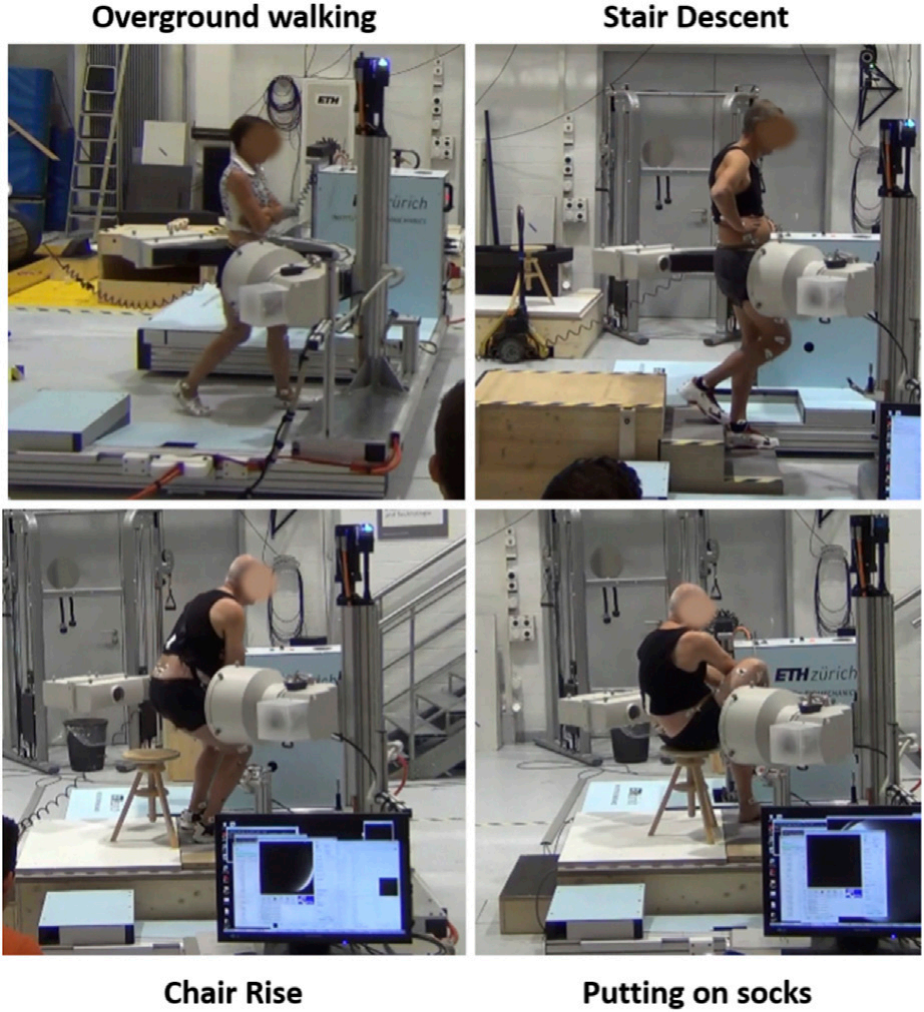
Synchronized moving video-fluoroscopy and motion capture measurement of activities of daily living.

Each subject was measured with synchronized moving video-fluoroscopy and optical motion capture at a reference standing upright position and while performing four ADLs: overground walking, stair descent, chair rise, and putting on socks (Figure 1). Given the overall limitation on allowable radiation exposure, determined by the overall imaging time, and imaging angle for a desired accuracy (D’Isidoro et al., 2017), no more than four activities could be assessed. Five cycles per activity were chosen for analysis, based on the image quality. Force plates on the ground and on the stair steps were used to identify cycle events. Cycles were defined for gait between two consecutive right heel strikes, for stair descent between the right toe landing on the step and the following right heel strike on the ground, for chair rise from beginning of trunk flexion to standing position. Gait was performed with the arms crossed in front of the torso, to allow an unobstructed view of the hip for video fluoroscopy. It has been shown that even a partial weight-bearing constraint of the arms does not influence the motion patterns at the joints of the lower extremities during gait (Stephenson et al., 2010). Putting on socks was performed in a seated position until maximum hip flexion. Prior to each experiment, each subject was given sufficient time to get accustomed to the moving fluoroscope without being exposed to radiation. Measurements were done once the moving pattern of the patient was deemed consistent and unaffected by the presence of the machine.

### 2.2 Video-fluoroscopy measurement

The moving fluoroscope consisted of a modified video-fluoroscopy C-arm (BV Pulsera, Philips Medical Systems) mounted onto a motorized trolley that allows horizontal tracking up to 5 m/s velocity and 9 m/s^2^ acceleration, and vertical tracking up to 1.33 m/s velocity and 4 m/s^2^ acceleration. Tracking is achieved by positional feedback of the subject’s joint using a wire sensor and a digital goniometer; the wire sensor is attached with a Velcro-strap to the waist of the subject for tracking the hip.

Specifically for the hip joint measurements, the C-arm was oriented such that the irradiation angle was 45° from the posterior side according to the guidelines described in a previous study for optimized accuracy of 2D/3D registration and low radiation dose delivered to the patient (D’Isidoro et al., 2017). Fluoroscopic images were acquired during motion at 30 Hz, with a standalone charge-coupled device camera (camera: IMPERX IPX-1M48-L, Imperx Inc., Boca Raton, United States; framegrabber: Matrox Solios eCL/XCL-B, Matrox Electronic Systems Ltd., Quebec, Canada) to allow a shutter time of 1 ms and an image resolution of 1,000 × 1,000 pixels with a grayscale resolution of 12 bit. The fluoroscopy system was operated at 8 ms pulse width, 100–110 kV and 12 mA*s. Due to the optimized irradiation angle, measurements were performed with a total radiation exposure below 5 mSv over all motion trials for each subject.

### 2.3 Motion capture

Optical motion capture was performed simultaneously to single-plane video-fluoroscopy. A Vicon MX system (Oxford Metrics Group, UK) using twenty-six MX40 and T160 infrared cameras recorded at 100 Hz the positions of skin markers, that were attached to the pelvis and the thigh of the patient according to the investigational site’s marker-set for lower extremity assessments 28.

MC and VF were synchronized temporally with an external trigger and spatially with a calibration tool with known relative position between a grid of metal beads and fixed skin markers.

### 2.4 VF-based kinematics

**FIGURE 2:**
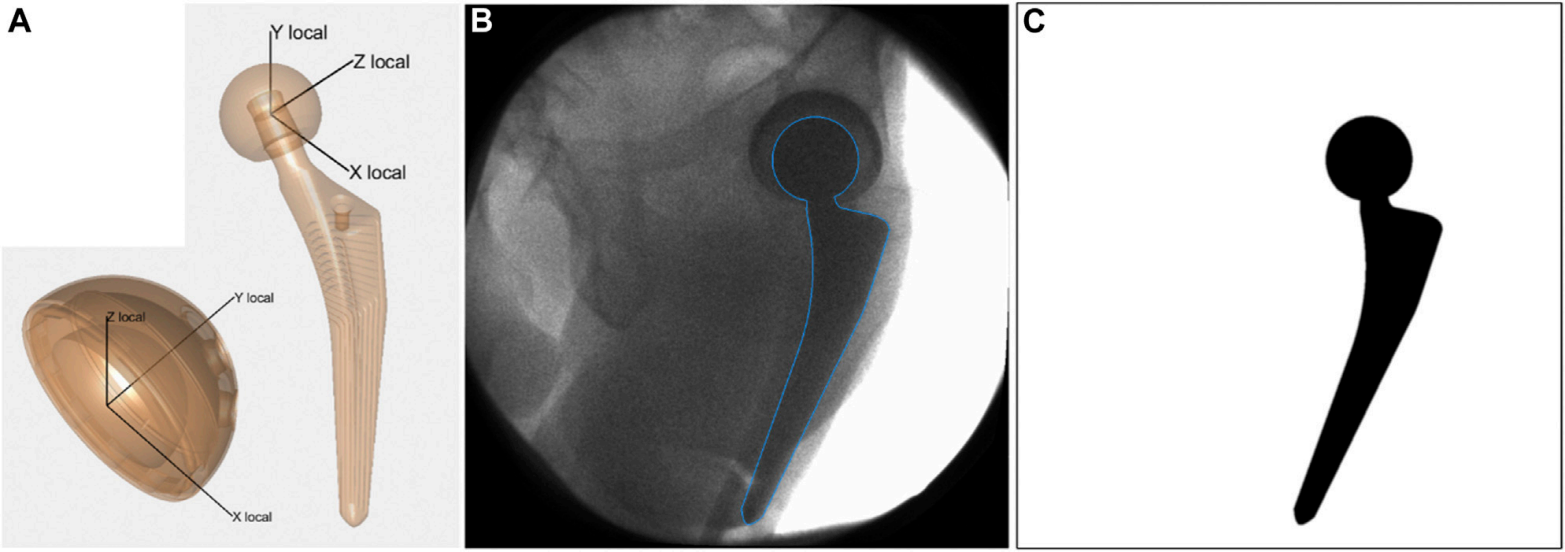
**(A)** Local coordinate systems defined for acetabular cup and femoral stem. **(B)** Acquired fluoroscopic image with overlay of registered femoral stem (blue line). **(C)** Simulated image of the femoral stem at the registered pose. It represents the simulated image that presented the highest similarity with the acquired fluoroscopic image, according to the gradient correlation similarity metric.

Local coordinate systems were defined for the computer aided design (CAD) models of the acetabular cup and of the femoral stem (Figure 2) obtained from the manufacturer. For each acquired fluoroscopic image, the corresponding 3D pose of each implant model was retrieved with an in-house developed semi-automatic 2D/3D registration procedure. Automatic registration minimizes the difference between an input real fluoroscopic image and simulated images at predicted poses of the 3D model. Simulated images of the implant are fast generated as binary images with no-shading rendering from VTK Python graphics library, and subsequently blurred with Gaussian filter to simulate a real imaging system (Figure 2). Similarity between the de-noised input image and the simulated images is computed with a gradient correlation metric. The evolutionary strategy optimizer from the NLopt library is used to find the optimum 3D pose of the implant for which the similarity metric is maximum.

**FIGURE 3:**
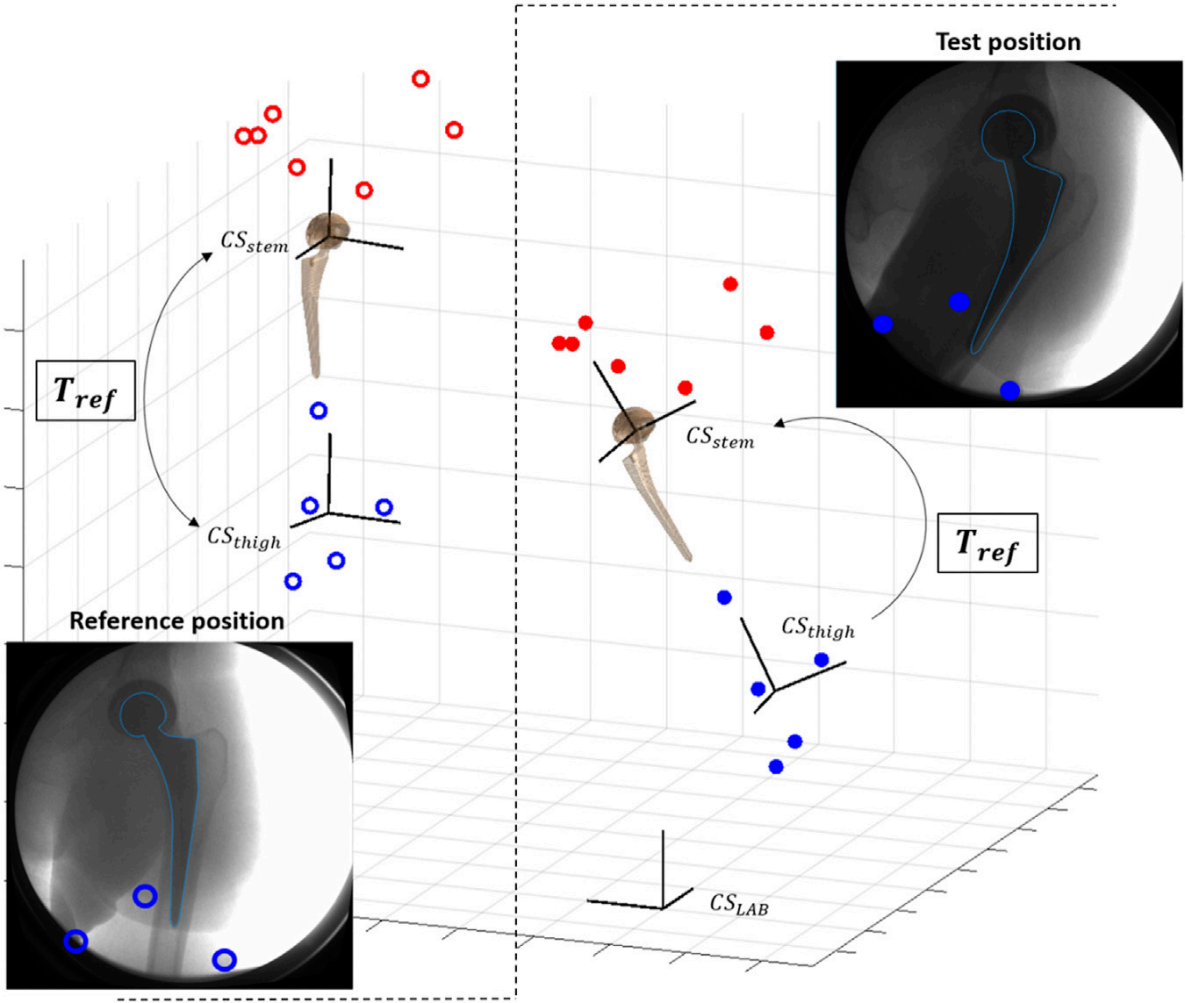
Scheme of pose initialization with skin markers (red for pelvis, blue for thigh): at reference standing position, the transformation *T*_*ref*_ between skin-marker based segment (*CS*_*thigh*_) and prosthetic component (*CS*_*stem*_) is computed. *T*_*ref*_ is used to initialize the pose of the prosthetic component for each new test position.

Convergence of the optimization to the optimum 3D pose was facilitated by providing a close initial guess that was derived from MC (Figure 3) in an automatic fashion. Reference skin marker-based segments of the hip joint were defined at the standing position, and their relative position with respect to the corresponding prosthetic component was calculated. This relative position at the standing position was used to estimate the 3D pose of the prosthetic components for each acquired fluoroscopic image from the 3D pose of the relative skin marker-based segment. The 3D pose for each acquired frame of the skin marker-based segments was computed by a least-square fit with the markers cloud at the reference standing position.

Since the acetabular cup is symmetric, its rotation around the symmetry axis cannot be uniquely determined by registration between image and implant model. This undetermined degree of freedom was then computed from the skin marker-based initialized value.

After automatic registration, manual correction of the 3D pose was performed by a trained user using a graphical user interface. The final registered 3D poses expressed in the local coordinate system of each prosthetic component were transformed into the anatomical coordinate systems. These were defined for both components based on the skin marker derived location of the relative joint segment at the reference upright standing position and according to the International Society of Biomechanics recommendations 35. Finally, kinematics curves obtained from the sequence of registered poses were filtered using a fourth-order bidirectional low-pass Butterworth filter with a cutoff frequency of 6 Hz. Anatomical hip joint rotations were expressed as a ZXY Cardan sequence (Wu et al., 2002), corresponding to a sequence of consecutive flexion-extension (FE), adduction-abduction (AA) and internal-external rotation (IE).

The registration accuracy of single-plane fluoroscopy from a 45° irradiation angle was evaluated for the femoral stem in a previous study 34: RMS errors for FE, AA and, IE were 1.4°,1.2°,2.0° during simulated gait and 0.6°,0.8°,1.75° during simulated sitting down, respectively.

## 3 Results

The average speed of the 15 THA subjects was 1.13 m/s during level walking, 0.56 m/s during stair descent, while the average duration of the tasks of chair rise and putting on socks was 2.15 and 1.65 s, respectively (Table 1).

**TABLE 1:** Kinematic parameters extracted for 15 THA patients during four activities of daily living.

	Gait	Stairs	Chair rise	Socks on
Average speed [m/s] or duration [s]	1.13 m/s (0.19)	0.56 m/s (0.12)	2.15 s (0.391)	1.65 s (0.42)
Flexion [°]				
Mean ROM	41.10 (6.23)	28.27 (3.64)	93.35 (8.89)	53.02 (9.65)
Mean Peak	32.3 (8.37)	39.29 (8.73)	100.68 (13.36)	106.41 (12.3)
Mean Min	−8.7 (10.45)	11.11 (8.60)	7.56 (10.51)	53.33 (14.32)
Average Mean	14.33	22.01	61.89	80.56
Inter-patient dev	8.98	8.24	11.48	12.13
Intra-p mean dev	1.93	2.32	7.17	3.94
Adduction [°]				
Mean ROM	10.12 (2.22)	15.85 (3.836)	10.37 (2.08)	16.86 (4.10)
Mean Peak	6.9 (4.40)	10.29 (4.21)	5.85 (3.27)	11.0 (9.54)
Mean Min	−3.14 (3.32)	−5.46 (4.35)	−4.35 (3.27)	−5.24 (8.78)
Average Mean	2.22	2.76	1.03	1.69
Inter-patient dev	4.01	4.06	3.51	9.41
Intra-p mean dev	1.43	1.84	1.79	1.72
Internal rotation [°]				
Mean ROM	11.80 (2.90)	16.28 (4.03)	13.37 (3.30)	24.88 (5.68)
Mean Peak	1.75 (5.73)	2.18 (7.46)	3.26 (4.61)	−0.6 (6.85)
Mean Min	−9.98 (5.82)	−14.02 (7.17)	−9.97 (4.43)	−24.43 (7.52)
Average Mean	−3.92	−5.42	−3.12	−13.9
Inter-patient dev	5.59	6.56	4.21	8.24
Intra-p mean dev	2.04	2.91	2.26	1.95

Value in brackets indicate standard deviations. “Inter-patient dev” is the mean standard deviation calculated over the mean cycles for the 15 THA patients, while “Intra-p mean dev” is the average of the mean standard deviations calculated per patient over the five cycles per activity.

**FIGURE 4:**
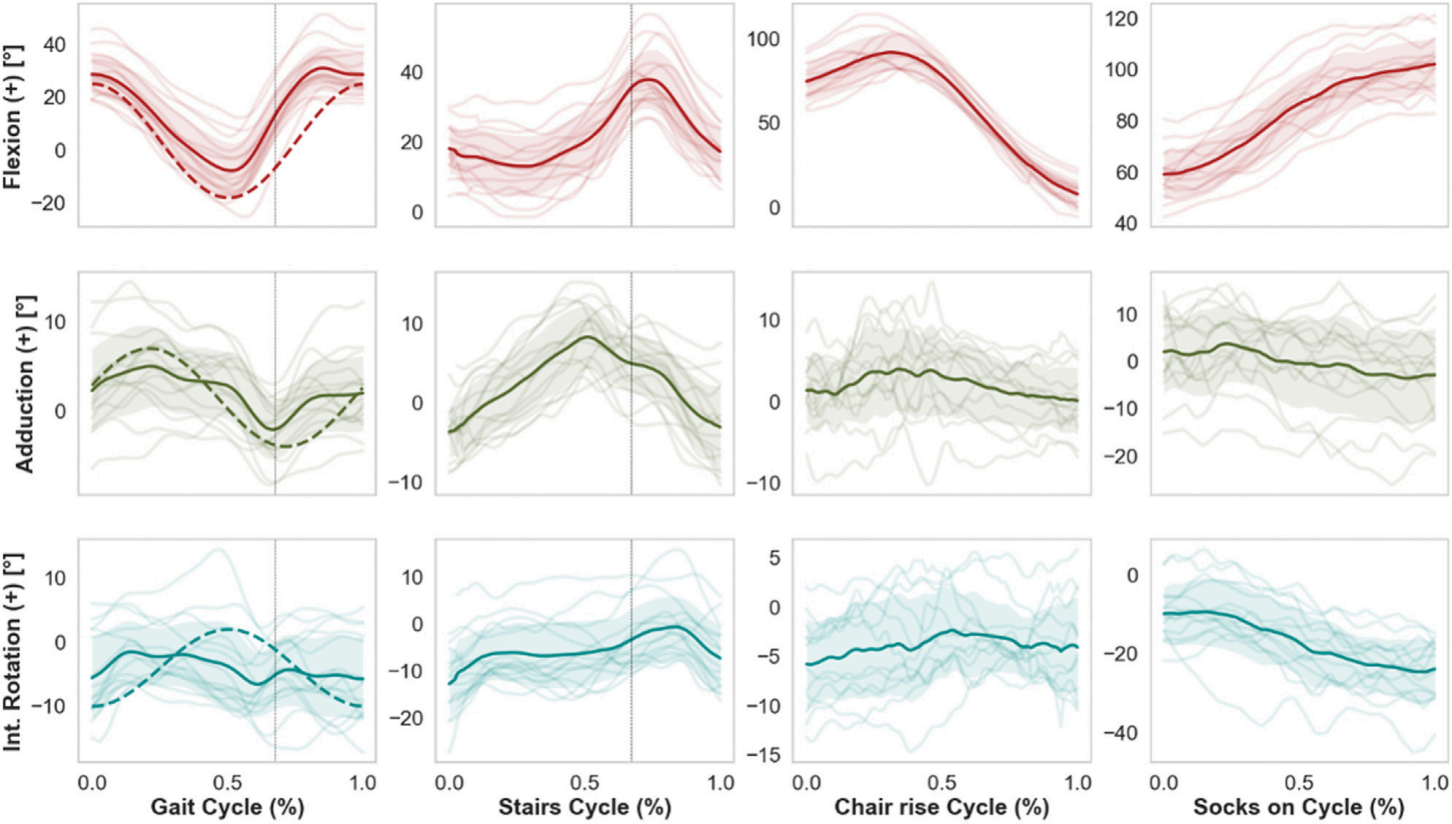
Mean kinematic curves (solid line) of 15 THA subjects during whole cycles of gait, stair descent, chair rise, and putting on socks. Shaded bands indicate standard deviations, while single semi-transparent lines refer to the individual per-subject kinematics averaged over five cycles. Dashed vertical lines indicate the time of toe-off for the kinematics of gait and stairs descent. Dashed curved lines superimposed on the activity of gait refer to the current ISO standard. Of note, the current ISO standard for wear testing fails to capture, e.g., the extensive range of flexion observed in specific daily activities such as chair rise or putting on socks, therefore these less frequent but potentially challenging activities are excluded from standard pre-clinical device tests.

During overground gait, the mean range of motion (ROM) was 41.1°, 10.1°, and 11.8° for FE, AA, and IE, respectively. The hip joint remained for the most part flexed, externally rotated and slightly adducted throughout the gait cycle (Figure 4), reaching extension up to a mean of 8.7° at late stance phase (ca. 56% gait cycle). The highest average hip flexion was 32.3° at terminal mid swing phase (ca. 85% gait cycle). The average external rotation was 3.9°.

Compared to gait, mean ROM for stair descent was smaller for FE and AA (28.3°, 15.9°, respectively) and larger for IE (16.3°). The average peak flexion was 39.3° at mid-swing phase of the descent (75% cycle). The hip reached higher peak adduction levels (10.3°) compared to gait around the toe-off. No particular trend was observed for IE, with average external rotation of 5.4°.

Starting from the seated position, the THA joint reached average peak flexion values of 100.7° (±13.36) for the sit-to-stand and 106.4° (±12.3) for the task of putting on socks, corresponding to average ranges of FE of 93.4° and 53.0°, respectively. Putting on socks was performed at average external rotation of 13.9°, with peak values of 24.4°.

For gait, average standard deviations from the mean cycles over all 15 subjects were 9.0°, 4.0°, and 5.6° for FE, AA and IE, respectively. Compared to gait, inter-subject variability was similar for stair descent, while larger for the FE during chair rise and putting on socks (Table 1). Intra-subject variability over the five cycles per activity was generally below 3° on average for all anatomical rotations and for all motion tasks, with larger values of mean standard deviations for the flexion during chair rise and putting on socks.

No hip separations or impingement were observed for any of the 15 THA subjects during the measured activities.

## 4 Discussion

This study is the first to the authors’ knowledge to measure with moving video-fluoroscopy the *in-vivo* THA kinematics during both routine and challenging activities of daily living performed in an unconstrained environment. The retrieved set of soft tissue artefact-free kinematics for 15 THA patients during four ADLs has been made publicly available (see Data Availability Statement). The observed patient-specific motions differed from the standard patterns applied for wear testing, supporting the statement that current pre-clinical wear test procedures do not reflect the experienced *in-vivo* daily motions and loadings and do not capture possible adverse loading scenarios such as separation, edge loading or hip impingement. The pre-clinical testing standards (e.g., ISO) allow a reliable comparison between devices in controlled conditions, and may indeed provide a reasonable estimate of long-term wear over millions of cycles. However, the unique kinematic patterns observed in the present study for specific activities may be quite important for short-term phenomena related to fluid film response within the joint over a single loading cycle, or single adverse events causing joint subluxation.

Studies have pointed out the scarce amount of motion analyses for challenging activities that could contribute more to wear than gait (Kolk et al., 2014). The activity of stair descent was associated with the highest peak hip contact forces (260% BW) among several daily activities analyzed by Bergmann et al. (2001) for four patients with instrumented implants. Standing up is performed by older adults 65 times a day on average (Egerton and Brauer, 2009), and sitting was reported to be the most frequent daily activity from analysis over 31 THA patients (Morlock et al., 2001). Chair rise is challenging both for maintaining balance and for producing the force needed to complete the movement starting from a high degree of hip flexion (Lunn et al., 2016). Putting on socks is a challenging daily activity for patients with a hip implant and the ability to perform it is included in the Oxford Hip Score questionnaire to evaluate THA outcomes. Accurate measurement of hip kinematics with skin marker-based motion capture during high range of motion activities, such as stand up or putting on socks, is hampered by at least two factors. The skin deformation due to the large amount of soft tissue surrounding the hip joint contributes to soft tissue artefact. Secondly, the limited markers visibility due to the flexion of the trunk with respect to the lower limbs may reduce accuracy in motion retrieval. This study showed that moving fluoroscopy represents a valid solution for accurate kinematic analysis of the hip joint during challenging motion tasks besides gait.

The retrieved mean curves and ranges of motion of hip angles during level walking differed from those of the current ISO standard patterns for wear tests (ISO 14242-1:2014, 2014), with an offset towards a more extended hip throughout the cycle in the ISO standard. Moreover, the observed inter-subject variations (average standard deviations of 9.0°, 4.0°, 5.6° from the mean FE, AA, and IE rotation, respectively) indicate considerable deviations of the individual motions from the mean gait curves. We point out that the observed variations among patients stem solely from different motion patterns and not from different implant orientations, since kinematics were described based on anatomical coordinate systems. While the ISO pattern may approximate the gait motion of the mean THA population, wear predictions based on average profiles as input do not necessarily correspond to effective wear generated by the patient-specific kinematics (Cook and Robertson, 2016). The effect on wear of the individual motion paths of the femoral head articulating against the acetabular cup was discussed in several studies (Gao et al., 2011; Mellon et al., 2013). Some related the rate of polyethylene wear with the degree of multidirectional motion and the associated cross shear (Bragdon et al., 1996; Bennett et al., 2000; Budenberg et al., 2012), which are not realistically captured by the ISO pattern according to our results.

The angle curves retrieved for the other activities of daily living were different than the gait ISO profiles (Figure 1). Higher ranges were computed for AA and IE during stairs descent, and for FE and IE during chair rise. Given these differences, the present dataset of patient-specific kinematics may be used in combination with loading profiles to investigate the effect on wear of a range of daily activities broader than the current ISO standard. Moreover, a single representative daily cycle may be defined based on a combination of these activities and on their frequency, and the importance of the sequence of motions for wear can be further investigated.

A similar dataset was proposed by Fabry et al. (2013), based on the *in-vivo* kinematics and kinetics of four THA patients measured by Bergmann et al. (2001) with skin-marker motion capture and instrumented implants. Fabry et al. generated mean motion and loading profiles for walking, knee bending, stair climbing, and chair rising, and combined those into a single motion sequence that was suggested for wear tests under more comprehensive conditions. The mean profiles were produced from the measurements of only four patients, thus they cannot be generalized. Compared to the present study, the mean ranges of FE for level walking and for chair rise were almost 10° and 32° smaller, respectively. Reduced mean range of FE by 11° during chair rise was also reported by Lamontagne et al. (2012) in a motion capture study of 20 THA patients. These differences may be partly due to the less accurate motion analysis technique. In fact, a recent study by Fiorentino et al. (2017) evaluated the effects of soft tissue artefact on hip angles and reported statistically significant underestimation of the hip flexion and of the range of FE by skin marker-based technique, which we have also observed in our own ongoing measurements (D’Isidoro et al., 2020). In these measurements, during dynamic activities the hip joint appeared less flexed, more adducted and more internally rotated with motion capture, compared to video fluoroscopy. Overall errors for motion capture were on the order of 7%–11% of total ROM (D’Isidoro et al., 2020). The theoretical registration accuracy of single plane video fluoroscopy has been estimated in (D’Isidoro et al., 2017), ranging from RMS errors of sub-1° up to 2°, depending on imaging angle. For the oblique imaging angle in the present study, errors of up to 0.5° could be expected. The range and motion profiles reported by Lamontagne et al. (2011b) for stair descent are similar to those of the current analysis.

Since no previous fluoroscopy study of the hip was carried out for the four daily activities described here, comparison could only be performed between the overground gait presented in this research and the treadmill gait analyzed with dual-plane fluoroscopy by Tsai et al. (2014) (28 subjects) and Fiorentino et al. (2017) (11 subjects). While overall motion patterns were similar, some differences were found. Tsai et al. reported the prosthetic joint to remain flexed, adducted and externally rotated throughout the treadmill gait cycle. While a similar range of FE was found in the present study, the hip attained extension up to a maximum of 19.3° at terminal stance of the overground gait, indicating a more upright walking posture of the subjects. This discrepancy may originate from the higher walking speeds achieved with the moving fluoroscope (1.13 m/s versus 0.77 m/s), the different patient population, or the use of skin markers rather than bony landmarks for definition of the anatomical coordinate systems. Another reason may be the difference between treadmill and overground gait. However, kinematic equivalence between these two activities was found in a previous study (Watt et al., 2010). Furthermore, hip extension up to 20° was also observed in the analysis of Fiorentino et al., where ROM for flexion was even higher than in the present study.

Reduced ROM of implanted hip joints compared to healthy ones was reported during overground gait in a few motion capture-based studies (Perron et al., 2000; Bennett et al., 2008) and confirmed in the present one. For the fifteen measured subjects, the mean speed and range of hip FE and AA were lower compared to normal elderly subjects measured for a large cohort by Bennett et al. (2008); likewise, the range of hip FE, and the peak hip extension, adduction and internal rotation were smaller compared to the twenty healthy subject measured by Beaulieu et al. (2010). The latter study suggested that THA patients walk with reduced flexion and adduction in order to produce lower moments to stabilize the pelvis in the sagittal and the frontal plane, respectively. They speculated that this altered gait patterns result from a weakness of the hip flexors and adductors, which was developed either pre-operatively as a result of disuse atrophy to avoid pain or post-operatively as a result of surgery (Lamontagne et al., 2012).

For the activity of stair descent, the range and the peak values of hip FE observed in the present study were smaller compared to those of healthy subjects from the study by Lamontagne et al. (2011b); this supports the finding that THA patients flex their operated leg to a minimal degree necessary to clear the step, a tendency that might be a result of reduced activation of hip flexors. However for the chair rise activity, the reduction in hip sagittal plane range of motion and peak hip flexion of THA patient with respect to healthy controls was not observed in this study, where range of hip flexion was even higher than the one of healthy subjects reported by La Montagne et al. This higher range of hip flexion might be explained from a larger forward lean required to initiate the chair rise due to reduced force generating capacity.

No hip separations or impingement could be detected in the present study. Accuracy of single-plane fluoroscopy in retrieval of in-plane translations is comparable to accuracy of dual-plane techniques (Tsai et al., 2013), with estimated errors smaller than 0.47 ± 0.38 mm (mean and standard deviation) for the femoral stem and smaller than 0.84 ± 0.64 mm for the acetabular cup. Therefore, at least the in-plane components of the relative movement between the hip implant segments can be reliably considered smaller than 1 mm. Other dual-plane (Tsai et al., 2014) and single-plane (Glaser et al., 2008) fluoroscopy studies also reported small hip separation during gait (<0.72 and <0.9 mm, respectively), supporting the statement that only minimal “hip pistoning” occurs during the swing phase of gait. It was speculated that co-contraction may be responsible of the limited hip separation (Park et al., 1999; Tsai et al., 2014). In contrast, other studies reported larger hip separations during gait (2.8 mm) and hip abduction/adduction (3.0 mm) (Lombardi et al., 2000) and during pivoting activity (3.3 mm) (Blumenfeld et al., 2011). The results from the present study suggest that no relevant hip separations occur for stair descent, despite the longer leg trailing phase compared to gait, as well as for higher range of motion activities such as char rise and putting on socks. However, the accuracy of the present study did not allow to characterize micro-separation during daily activities. Studies have highlighted the importance of introducing micro-separation in the wear tests, as it affects the lubrication regime and the wear rate of the hip implant (Beadling et al., 2016).

Besides the low out-of-plane accuracy intrinsic to single-plane fluoroscopy, another limitation exists. The rotational degree of the acetabular cup around its symmetry axis could only be retrieved from the skin markers locations, thus its accuracy is affected by the soft tissue artefact. The resulting error propagates mostly to the internal rotation of the hip joint that is overestimated by skin marker-based techniques by an average of 5.8° during dynamic activities (Fiorentino et al., 2017). Other studies registered the pelvis segment with a model segmented from a CT scan that had not been acquired for the cohort of this study, as CT scans are not part of the standard routine procedure for total hip replacements. On the other hand, the present study showed a novel way to register the pelvis pose of an implanted hip without the need of a CT scan, thus with a considerable lower radiation dose delivered to the subjects at the expense of a lower accuracy. Another novelty was the use of skin markers to provide an initial guess of the 3D pose of the hip joint for each acquired fluoroscopic frame; this sped up the lengthy 2D/3D registration process and has not been reported in literature up to the author’s knowledge. Finally, no previous study analyzed the activity of putting on socks for THA patients.

In conclusion, accurate *in-vivo* patient-specific kinematics of a sample THA population during four daily activities were produced and made publicly available. The importance of using individual patterns was highlighted by the inter-subject variability observed in the present study for different activities of daily living. The presented dataset can help to gain a better understanding of the *in-vivo* mechanisms of implant impingement and wear and could potentially improve the current range of motion and wear predictions performed with computational and laboratory simulators.

## Data Availability

The datasets presented in this study can be found in online repositories. The names of the repository/repositories and accession number(s) can be found below: https://www.research-collection.ethz.ch/handle/20.500.11850/404705.
